# Supplementary figures and images for: Long-Term Results of Pediatric Liver Transplantation for Progressive Familial Intrahepatic Cholestasis
Source: J Clin Med. 2022 Aug 11;11(16):4684. doi: 10.3390/jcm11164684 (PMC9410346; doi:10.3390/jcm11164684)

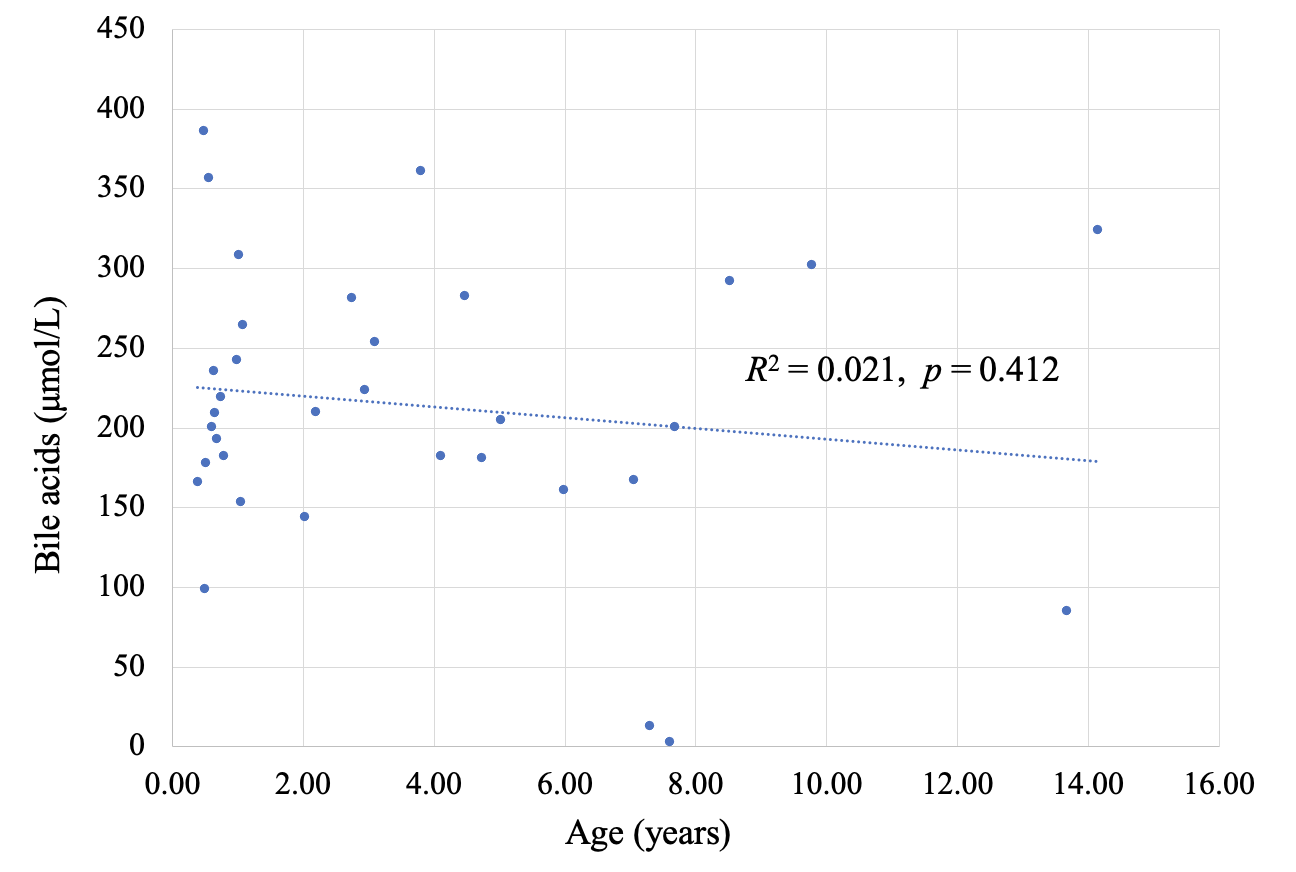

Supplement: Supplementary file 1 [file jcm-11-04684-s001.zip › jcm-1802822-Figure S1.png]
